# Variant of Lemierre's Syndrome Secondary to Trauma

**DOI:** 10.1155/2010/123943

**Published:** 2010-08-31

**Authors:** Sani M. Bukari, Renato Roxas, Deepak Kamat

**Affiliations:** ^1^Medicine-Pediatrics Detroit Medical Center, Wayne State University, Detroit, MI 48201, USA; ^2^Medicine Pediatric Program, Wayne State University, Detroit, MI 48201, USA; ^3^Pediatric Program, Children Hospital of Michigan, Detroit, MI 48201, USA

## Abstract

Classic Lemierre's syndrome is a septic internal jugular venous (IJV) thrombophlebitis secondary to oropharyngeal anaerobic infection in adolescent and young adult. Though upper respiratory tract infection is the most common antecedent, it has also been described following skin, soft tissues, genitourinary and gastrointestinal infections. Fusobacteria necrophorum is the commonest pathogen isolated from blood and tissue cultures but other bacteria like Eikenella correndens, Bacteroides melaninogenicus and Non Group A Streptococcal species have also been reported. The overall incidence of Lemierre's and Lemierre's like syndromes has declined since the first case report in 1936. There is however, a resurgence of cases in recent years due to more judicious use of antibiotics for treatment of upper respiratory tract infection among medical practitioners. The main stay of treatment of complete Lemierre's syndrome is prolonged antibiotic coverage and surgical drainage of nonresolving abscesses. Currently, there is no consensus opinion on the use of anticoagulation in patient with complete Lemierre's syndrome complicated by septic internal jugular thrombosis and embolism. High index of suspicion is required for early diagnosis of Lemierre's and Lemierre's like syndromes. Early and effective antibiotics therapy may prevent the development full spectrum of the syndrome and its associated complication.

## 1. Introduction

Classic Lemierre's syndrome is septic internal jugular venous (IJV) thrombophlebitis secondary to oropharyngeal infection. This usually occurs in adolescents and young adults and usually follows oropharyngeal anaerobic sepsis [1]. Though oropharyngeal antecedent infections are the usual source of classic Lemierre's syndrome, there have been reports of necrobacillosis following infection of skin and soft tissue, genitourinary and gastrointestinal system. By far the commonest pathogen isolated from blood and tissue cultures is *Fusobacteria necrophorum* but other organisms like *Eikenella corrodens, Bacteroides melaninogenicus,* and nongroup A Streptococcal species have also been isolated in some cases [1, 2]. We present a case of a 5-year-old male with Lemierre's-like syndrome secondary to pencil tip trauma to pharynx.

## 2. Case Report

A 5-year-old previously healthy male presented with pain, difficulty in swallowing and right-sided neck swelling. Four days prior to presentation, he sustained a pencil scratch trauma to the right oropharynx secondary to a fall on the bed whilst playing with a pencil in his mouth. He bled moderately and was taken to nearby emergency room where the bleeding stopped spontaneously. Examination at that time showed minor superficial mucosa trauma. The parents were reassured, and the patient was discharged home on Tylenol for pain. He drooled for a few hours but returned to baseline activity level on the same day. Three days later, he complained of right-sided neck pain associated with reduced oral intake. The parents noticed right-sided jaw fullness and swelling. He was sent to emergency room the next day upon the recommendation of his pediatrician. In the emergency department, he looked unwell with fever of 38.9°C, right neck and jaw swelling with multiple right cervical lymphadenopathy, the largest measuring 3 cm, with overlying erythema. He also resisted neck movements due to pain. The rest of the examination of oropharynx, ears, and nose as well as other systems were within normal limit.

Complete blood count showed white cell count of 13,000/mm^3^, with 84% neutrophils and 10% bands. However, blood culture was negative. Soft tissue radiograph showed asymmetric right neck swelling, deviation of airway to the left, without evidence of a foreign body. Computerized tomography [CT] scan of neck showed a lesion with air-fluid interface, consistent with an abscess, in the right parapharyngeal space with surrounding inflammatory changes. The inflammation extended into the parotid gland, carotid sheath, the masticator muscles, as well as the retropharyngeal space, with mild mass effect noted on the airway. The upper part of internal jugular vein could not be visualized, possibly due to compression or thrombosis of upper third of the internal jugular vein. Further imaging was done with magnetic resonance angiography and venography [MRA/MRV] which confirmed the above findings and also showed the absence of blood flow in the upper third of internal jugular vein due to compression from the adjacent soft tissue mass and abscess.

The patient was treated with intravenous ceftriaxone and clindamycin for five days and then discharged home on oral clindamycin to complete a 14-day course of antibiotics as per the recommendation of an infectious disease consult. Repeat CT scan of the neck on day 7 of treatment showed subtle residual asymmetry of soft tissue of neck without abscess formation and normal anatomy of the rest of tissues.

## 3. Discussion

Since the first comprehensive review of 20 cases of “human necrobacillosis” or Lemierre's syndrome by Lemierre in 1936, several variants and Lemierre's-like syndrome have been reported and described. Classic Lemierre's syndrome is an anaerobic oropharyngeal infection complicated by septic thrombophlebitis of the internal jugular vein. It typically occurs after an upper respiratory tract infection in adolescents and young adults [1, 2]. 

The overall incidence of Lemierre's and Lemierre's-like syndromes is low 0.9–2.3 cases per million per year in Europe (5, 6). The incidence is said to be higher between the ages of 16–23 years with male predominance [5]. The classic syndrome typically has three stages; starting with oropharyngeal or tonsilar infection, followed by extension of inflammation and infection into parapharyngeal and retropharyngeal space within one week after the infection [6]. The third and final stage of disease involves septic thrombophlebitis in the internal jugular vein with metastasis commonly to lungs, and occasionally to liver, spleen, bones, kidneys, and meninges with associated septicemia. [3, 4]. This carried a high mortality rate during preantibiotic era. The incidence of Lemierre's syndrome has declined dramatically since it was described in 1936 due to ubiquitous and early use of antibiotic to treat upper respiratory tract infections. There have been reports of resurgence of cases in recent years because of more judicious use of antibiotics among medical practitioners [4].

 Our patient, a 5-year-old male developed the oropharyngeal infection after a pencil tip injury to the pharynx. He presented with a deep pharyngeal tissue abscess associated unilateral neck swelling, fever, and reduced oral intake. We postulate that the injury could have resulted in inoculation of oral microflora, possibly Fusobacteria and other organisms, into the deep pharyngeal spaces and this lead to the development of abscess. Though our patient had negative blood culture and tissue culture was not done, there was clear evidence of severe infection from the toxic look of the child, neutrophilic leukocytosis, and CT scan findings of deep pharyngeal tissue abscess associated with compression of adjoining structures including internal jugular vein. The otolaryngology service was consulted to obtain tissue for culture. However, the procedure could not be performed due to the deep seated nature of the infection, making the procedure technically difficult. Moreover, the patient had very good response to broad spectrum antibiotic therapy. The occlusion of internal jugular vein, secondary to external pressure from the abscess rather than thrombophlebitis of classic Lemierre's syndrome, may represent an early stage in the pathogenesis of septic thrombophlebitis. The early diagnosis and effective antibiotic therapy in this case possibly prevented progression to thrombosis. With advancement of diagnostic technology and early effective antibiotic therapy, a full spectrum of Lemierre's disease may not occur. 

 The mainstay of treatment of Lemierre's syndrome is prolonged antibiotic coverage and surgical drainage of nonresolving abscesses. The diagnosis should be confirmed with both aerobic and anaerobic tissue and blood cultures. The choice of antibiotics should initially be broad spectrum and subsequently changed according to the sensitivity of microorganisms from tissue and blood cultures. Our patient received ceftriaxone and clindamycin intravenously followed by oral clindamycin to complete a 14-day course compared to the usual (3–6)-week course of antibiotics recommended for advanced Lemierre's syndrome. This is because our patient did not develop the third stage, septic thrombophlebitis and metastatic abscesses, which requires prolonged treatment.

Currently, there is no consensus opinion on the use of anticoagulation in a patient with complete Lemierre's syndrome with septic internal jugular thrombosis and embolisms. There is no available literature on randomized controlled trial to assess the efficacy of anticoagulation on septic venous thrombosis [7]. Bach et al [8]. reported one case of Lemierre's syndrome which did not improve until heparin was added to treatment on 3rd day. The use of anticoagulation should thus be individualized based on the peculiarities of each case.

 In conclusion, a high index of suspicion is required for the diagnosis of Lemierre's and Lemierre's-like syndromes. Early and effective antibiotic therapy may prevent the development of full spectrum of the syndrome and associated complications.

## Figures and Tables

**Figure 1:**
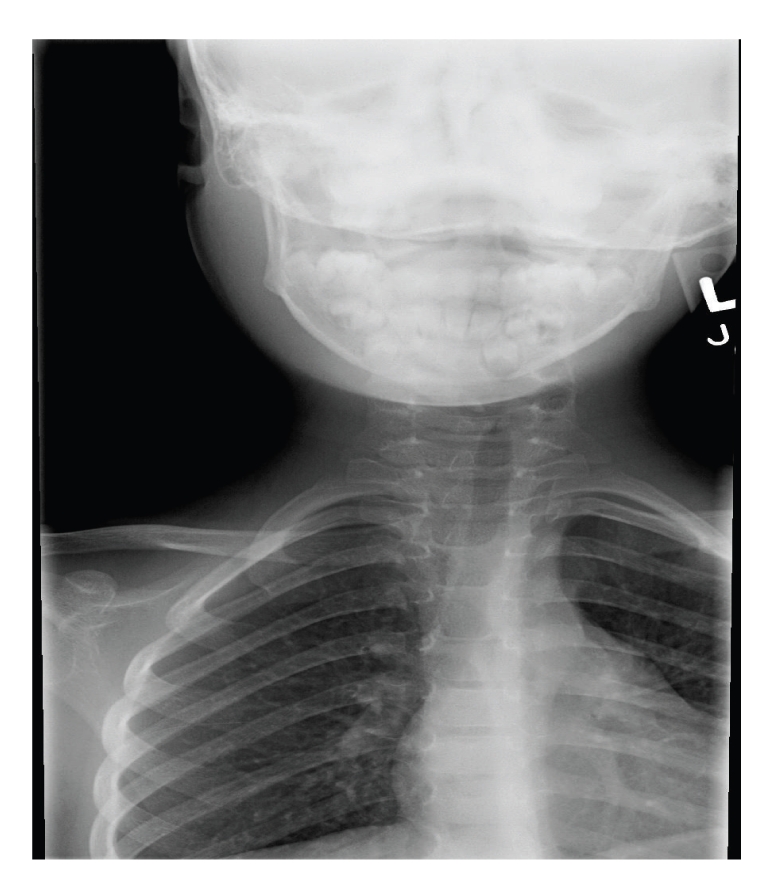
Soft tissue radiograph of the neck with deviation to the left and narrowing of upper airway.

**Figure 2:**
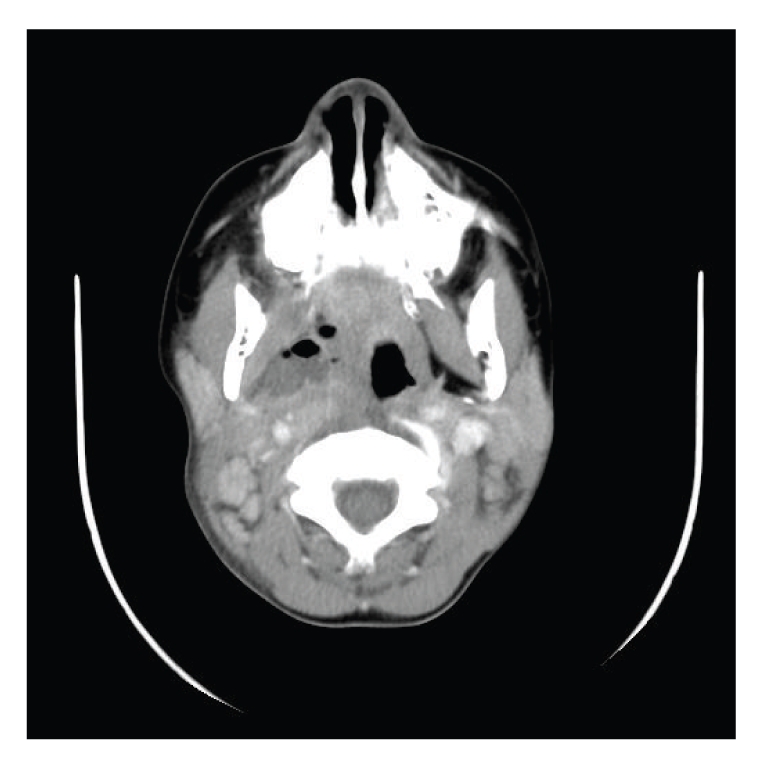
A CT scan with contrast showing compression of right internal jugular vein.

**Figure 3:**
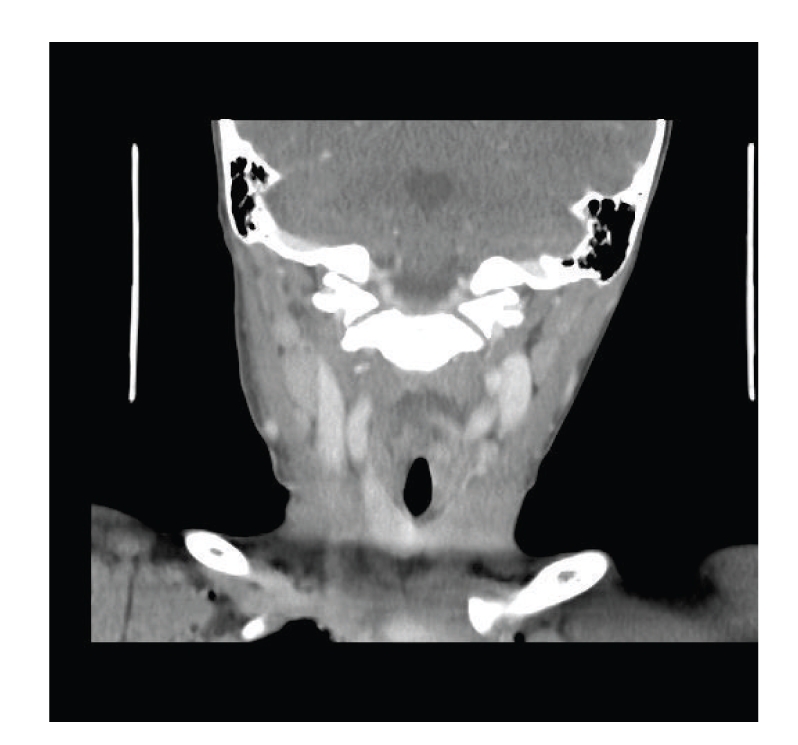
Magnetic resonance venogram showing compression of the upper part of the right internal jugular vein.
